# The Proper Treatment of Malarial Fever

**Published:** 1884-07

**Authors:** F. W. Ingalls

**Affiliations:** Kingston, N. Y.


### THE PROPER TREATMENT OF MALARIAL FEVER.

F. W. Ingalls, M. D., Kingston, N. Y.

[*Read before the Homœopathic Medical Society of New York.*]

The usual and popular treatment is to extinguish the intermittent paroxysm, or suppress them from time to time until by the use of some appropriate remedy they may be termed a cure.

How many patients we have in our communities that use Quinine almost every week during the entire season, to commence again as each spring returns, and again, and again a continued suppression called a cure!

It is a fact, however, that there are cases that to suppress from time to time is all we can do. Duty demands that we make the number very few, which we only can by giving remedies as they are indicated and not become routinists.

Epidemic intermittents demand more care, each case its own peculiar drug, as there is no doubt that some of this kind continually suppressed will leave the case to suffer from peculiar chronic ailments difficult to cure, and as difficult to name, unless we say in the stereotypic phrase of the day, "Oh! you are full of malaria."

*　　*　　*　　*　　*　　*　　*

There is no doubt but that Quinine is in some cases the perfect simillimum and is the drug we should be most familiar with. As it is among, if not the very first, drug that called into practical existence the law of *similia*, we should know it as a curative as well as a palliative drug.

As physicians, our knowledge of palliatives or palliative treatment should be complete—should go hand in hand with the pure science of *similia*. A continual study of materia medica will increase the number of palliative as well as curative drugs. It is to be hoped that we may in this Society to-day hear of many new modes of palliation, as well as cure, of this disease.

Hahnemann says that the remedy employed is to be selected from medicines hitherto tried, commonly from the non-antipsorics, shall likewise (as the surest means), be able to excite in healthy persons two (or all three) of the morbid stages that are similar; or, at least, it shall have the faculty of exciting, with all its accessory symptoms, the strongest and most prominent of these two or three consecutive stages. Yet the state of the patient during the apyrexia, especially must indicate the choice of remedy.

To administer the remedy immediately or very shortly after the termination of the paroxysm, as soon as the patient has, in some measure, recovered from it. Administered in this manner it has sufficient time to produce in the organism all its various effects, to restore health without violence or commotion; whereas, if taken immediately before the paroxysm (even though it were homœopathic or specific in the highest degree), its effect would coincide with the renewal of the natural disease and excite such a strife in the organism, so powerful a re-action, that the patient would lose at least a great portion of his strength, and even life would be endangered.

If the apyrexia be of short duration administer the remedy as soon as the perspiration, or other symptoms, pointing out the termination of the paroxysms, begin to diminish.

When a single dose of the appropriate remedy has destroyed several paroxysms, and manifestly restored health, and notwithstanding which, indications of a fresh attack are seen some time after, then only ought the same remedy to be repeated, provided the totality of the symptoms is still the same. But this return of the same fever after an interval of health is not possible, except when the cause which excited the malady, in the first instance, still exercises its influence upon the convalescent, as occurs in marshy countries.

In such a case a permanent cure is seldom effected, but by removing the patient from this exciting cause, and advising him to go and reside in a mountainous district, if that which attacked him was a marsh intermittent fever.

As almost every medicine produces a peculiar fever, even a species of intermittent, consequently there are an immense number of medicinal substances to cure intermittents.

When a remedy is found to be specific in an epidemic of intermittent fever, and there is no marshy influence to oppose its operation, then the obstacle generally arises from the psoric miasm; consequently antipsoric medicine ought to be employed until health is perfectly restored.

Allow me to refer all to a re-reading of the ideas given by Hahnemann and a renewed trial of the same.

A perfect choice of the remedy will cure the case before a complete paroxysm of the disease, if one can make that choice.

There is no use of referring you to the materia medica to aid in that choice; you all know where to find it.

To increase a renewed study of the same, and throwing aside the routine practice of treating the name of a disease with a popular remedy, and after failure in the trial of the same to resort to that study, is only to make more work and less honor to ourself and the profession.

The scientific treatment given by Hahnemann requires study and work, and it will the better fit us to care for all that come. To give each case that study will make the treatment thereof most scientific, and prove to the world that there is a science in the treatment of the sick. The time is not far distant when the people will demand that careful treatment that science only can give. Close and careful work on our part will give it them. Now is the time to do it, and one and all must be up and at it, no delay, but study as close as Hahnemann's will turn the intermittent to us for cure. Such study will stamp the name of science and scientific upon the practice of medicine as never before, and we will receive due honor in placing a knowledge of the second best gift of God to man in the minds of every one.

